# Relevance of the Anti-Inflammatory Properties of Curcumin in Neurodegenerative Diseases and Depression

**DOI:** 10.3390/molecules191220864

**Published:** 2014-12-12

**Authors:** Yousef Tizabi, Laura L. Hurley, Zakiya Qualls, Luli Akinfiresoye

**Affiliations:** Department of Pharmacology, College of Medicine, Howard University, Washington, DC 20059, USA; E-Mails: laura.hurley@students.mq.edu.au (L.L.H.); quallszm@njms.rutgers.edu (Z.Q.); lra28@georgetown.edu (L.A.)

**Keywords:** curcumin, inflammation, depression, Parkinson’s disease, neuroprotection

## Abstract

This review is an attempt to summarize our current understanding of curcumin’s potential as a neuroprotectant and an antidepressant. This dual property confers a unique advantage to this herbal medication, believed to be devoid of any major side effects, to combat commonly observed co-morbid conditions of a neurodegenerative and a neuropsychiatric disorder. Moreover, in line with the theme of this series, the role of inflammation and stress in these diseases and possible anti-inflammatory effects of curcumin, as well as its interaction with signal transduction proteins as a common denominator in its varied mechanisms of action, are also discussed. Thus, following a brief introduction of curcumin’s pharmacology, we present research suggesting how its anti-inflammatory properties have therapeutic potential in treating a devastating neurological disorder (Parkinson’s disease = PD) and a debilitating neuropsychiatric disorder (major depressive disorder = MDD). It is concluded that curcumin, or better yet, an analog with better and longer bioavailability could be of important therapeutic potential in PD and/or major depression.

## 1. Curcumin

Curcumin, a diarylheptanoid, is the principal curcuminoid of the popular South Asian spice turmeric (*Curcuma longa*), which is a member of the ginger family (Zingiberaceae). Curcumin and turmeric’s other two curcuminoids, desmethoxycurcumin and bisdesmethoxycurcumin are natural phenols responsible for the yellow color of turmeric. Indeed, because of its bright-yellow color, curcumin is used as a food coloring as well as food additive. Curcumin can exist in several tautomeric forms, however, the enol form is more stable in the solid phase and in solution [1].

Curcumin’s antioxidant [2,3,4], hepato- and nephroprotectant [5,6,7], antimicrobial [8,9], anti-inflammatory [10,11,12,13] and potential anti-depressant properties [14,15,16,17,18,19] are well documented. Epidemiological studies have demonstrated that societies that widely use curcumin show reduced incidence of inflammation-influenced and cognitive function diseases such as Alzheimer’s disease [20,21,22,23]. It has also been suggested that curcumin may reduce the incidence of Parkinson’s disease (PD), as some studies have shown an absence of age-related changes in nigral dopaminergic neurons in Indian populations that consume large amounts of curcumin [24,25,26]. Moreover, as discussed below, numerous *in vitro* and *in vivo* studies provide substantial evidence for a protective effect of curcumin against insults that may precipitate PD-like symptoms.

## 2. Depression

### 2.1. General Considerations

Major depression is a disorder with many definitions and manifestations and with a 12-month prevalence rate of 6.3% to 10.3% in Western societies [27]. Symptoms may vary, but often include anhedonia, disrupted sleep patterns, lack of motivation, or emotional distress (e.g., anxiety). Although a number of clinically effective treatments are available, a large segment of patients are non-responsive (*i.e.*, exhibit treatment-resistance to first-line interventions: [28,29]). Recent discoveries provide growing support for presence of cellular atrophy and neuronal death in major depressive disorder (MDD) as well as neurotrophic effects associated with antidepressants [30,31,32,33,34]. Thus, converging evidence support neurotrophic effects as a unifying hypothesis for antidepressants efficacy in treating MDD (see [35] for a detailed review). Moreover, activation of the ERK⁄MAPK pathway by antidepressants increases the expression of nuclear CREB, which facilitates the expression of neurotrophic/neuroprotective proteins such as Bcl-2 and brain-derived neurotrophic factor (BDNF). A current hypothesis posits that curcumin alleviates depressive behavior through activation of ERK-Bcl-2-BDNF neurotrophic pathway [36,37,38,39,40], particularly in areas implicated in the pathophysiology of depression (e.g., hippocampus and olfactory system: [41,42,43]). 

### 2.2. Depression-Inflammation

The purpose of inflammation is primarily to remove or inactivate potentially damaging agents or damaged tissues. This response is mediated via one of two cell systems: glia of the central nervous system (CNS), and lymphocytes, monocytes, and macrophages of the hematopoietic system [44,45]. Neuroinflammation is the brain’s response to injury, infection, or disease. Although, initial immune or inflammatory response is to remove damaged tissue or to inactivate potentially damaging agents, over-activation of the system can have severe detrimental consequences including precipitation of depressive-like behavior. Thus, patients who have major depressive disorder show alterations in immunologic markers such as increases in pro-inflammatory cytokines (discussed below). Moreover, chronic low-grade inflammation may result in changes in brain structure and synaptic plasticity leading to neurodegeneration [46,47,48,49]. This, coupled with a reduction in neuroprotection, may not only exacerbate depression, but may also lead to dementia, particularly in older people [48,50]. Furthermore, research suggests that neuroinflammation is suppressed by norepinephrine (NE), and that NE uptake inhibitors’ therapeutic efficacy in depression may be at least partially related to this mechanism [51]. Interestingly, Interferon-γ (IFN-γ), a cytokine that is critical for innate and adaptive immunity, induces the enzyme indoleamine 2,3-dioxygenase (IDO), which causes reduction in tryptophan availability, leading to a reduction in serotonin synthesis in the brain that has been implicated in depression [52]. 

## 3. Cytokines

Cytokines are a large family of small signaling proteins secreted from various cell types that elicit varied biological activities including induction of both anti- or pro-inflammatory responses. Anti-inflammatory cytokines are generally released to counteract the pro-inflammatory cytokines. This regulation helps limit damaging effects of prolonged or excess inflammation caused by the pro-inflammatory cytokines. However, cytokines dysregulation can lead to insufficient mediation or inhibition of normal immune reaction leading to disease manifestation including neurodegenerative diseases (e.g., PD) as well as major depression [53,54,55,56,57,58]. For example, interleukins (IL-1, IL-6), and TNF-α are released in response to various toxins such as lipopolysaccharide (LPS: a compound derived from membrane of gram-negative bacteria causing inflammatory-mediated damage) and typically cause a low-level inflammatory response to combat the insult [59,60]. However, these cytokines tend to have dual effects. For example, IL-6 may not only have a pro-inflammatory action [61,62], but may also act to attenuate or down-regulate synthesis of other pro-inflammatory cytokines (e.g., IFN-γ, IL-1, and TNF-α) [59,63,64]. IL-1, and TNF-α, as mentioned earlier also have acute pro-inflammatory actions, and dysregulation of production of one or more of these cytokines may precipitate a variety of neurological disorders including PD as well as MDD [65,66,67,68]. The mechanism of cytokine actions include impairment of neurogenesis and exacerbation of neuronal death [69,70,71,72,73] and may involve disruption of survival signaling pathways and caspase-dependent cascades, as well as alteration of normal receptor function [71,72,73].

## 4. Parkinson’s Disease

### 4.1. General Considerations

Parkinson’s Disease (PD), the second largest neurological illness in the elderly after Alzheimer’s, may affect as many as 6.3 million people worldwide. This neurodegenerative neurological movement disorder mostly affects people over the age of 65, however 15% of people can develop it before the age of 50. PD is a progressive neurodegenerative disorder that causes increased debilitating symptoms resulting from loss or damage to dopaminergic cells in the substantia nigra (SN). The causes for the development and progression of PD invariably involve an interaction between the person’s environment and genetic disposition. In fact, many studies link PD with exposure to various endogenous (e.g., salsolinol) and exogenous toxins (e.g., MPTP = 1-methyl-4-phenyl-1,2,3,6-tetrahydropyridine) (reviewed by [74]). Therefore, these compounds are often used to create *in vitro* and *in vivo* models to study PD. Salsolinol is an endogenous neuromodulator in dopaminergic cells formed during the metabolism of dopamine [75]. Dysregulation of salsolinol, especially its (*R*)-enantiomer form in the brain, is thought to contribute to development of PD [76,77,78,79]. It has been proposed that salsolinol and its derivatives (e.g., norsalsolinol, N-methylnorsalsolinol, N-methylsalsolinol) may serve as a marker for PD as they are increased in the cerebrospinal fluid [80] and the urine [81] of patients with PD. Exogenous compounds that are non-isoquinoline derivatives have also been shown to induce dopaminergic cell death in the SN. One such example is rotenone, a naturally occurring plant toxin that has been developed into a widely used pesticide and insecticide. Rotenone’s toxicity has been demonstrated in various *in vitro* [82,83,84] and *in vivo* [85] studies. Moreover, it has been shown that when low doses of multiple exogenous factors are combined, a synergistic neurotoxicity may result. Thus, combination of nontoxic or minimally toxic concentrations of rotenone and LPS can result in exaggerated or synergistic toxicity [86]. Recently, we have provided *in vitro* evidence that exposure to a combination of salsolinol and rotenone can have synergistic toxicity. Moreover, we showed that pretreatment with curcumin can protect against such toxicity [87].

### 4.2. PD and Inflammation

As mentioned above, the immune system is designed to help protect the body against disease, toxic agents, stress, and injury. Inflammation is the first response to infection and injury as the body initiates a defense, mediated by cytokines towards the healing process. In a normal functioning system, pro- and anti-inflammatory cytokines function in a regulatory loop to maintain the proper response. However, breakdown of the normal response by factors such as stress or other insults can cause inflammation to become persistent and harmful [88]. For example, inflammation induced by LPS causes long-term increase in brain TNF-α from microglia months after it has subsided in the periphery [89]. This increased pro-inflammatory response may also induce a delayed and progressive loss in dopaminergic neurons in the substantia nigra, similar to that seen in PD suggesting that enhanced neuroinflammation could lead to PD [89,90,91]. In addition, the elevated levels of pro-inflammatory cytokines (e.g., IL-6 and IL-1β) can induce symptoms of a syndrome termed “sickness behavior” [89,92,93], which include depression, reduction in locomotor activity, anhedonia, anorexia and cognitive disturbances [92,93,94,95]. In recent years, a number of findings have reinforced the idea that unregulated inflammation can lead to major depression and neurodegeneration. This contention is further supported by the findings that administration of high levels of pro-inflammatory cytokines can cause changes in behavior similar to depression and that attenuation of inflammatory response reduces depressive symptoms [96,97]. Thus, there is substantial support for inflammation as a common neurobiological substrate responsible for the co-morbid manifestation of neurodegenerative diseases (e.g., PD) and MDD. Curiously, it has been recently proposed that inflammation may also be the common mediator of commonly observed co-morbid conditions of depression and chronic pain [93]. 

## 5. Role of Stress

Stress is known to alter immune functioning [98]. Maes *et al* [98] were the first to show that psychological stressors in humans induce inflammatory responses through production of pro-inflammatory cytokines, such as IFN-γ and TNF-α. Subsequent studies have shown this to be true with a variety of other stressors as well [99,100]. Animal studies have also demonstrated that stressors increase cytokine levels such as IL-1beta and IL-6 in the blood and in various regions of the brain [49,69,101,102]. Goshen *et al.* [69] have specifically shown that after chronic mild stress normal mice show depressive-like behavior and an increase in IL-1β in the hippocampus. However, IL-1 receptor-deficient mice do not show such behavioral changes. Recently, it has been shown that direct administration of TNF-α can also induce a depressive-like state that can be blocked with the anti-TNF-α antibody [103]. Interestingly, it has been proposed that the action of some anti-depressants may also be attributed to a reduction in pro-inflammatory cytokines [97,104]. Moreover, neurological diseases like Alzheimer’s disease [50,105], amyotrophic lateral sclerosis, epilepsy, Huntington’s disease, multiple sclerosis, and PD [106] are often co-morbid with depression and express inflammatory markers. Thus, stress via enhanced release of pro-inflammatory cytokines such as TNF-α, IL-1β and IL-6 may not only precipitate depression, but may also accelerate the neurodegenerative processes. For this reason drugs that may interfere with detrimental consequences of stress on inflammatory pathways may be of therapeutic potential in neurodegenerative and/or mood disorders. 

Thus, direct effects of stress on inflammatory pathways, key proteins involved in signal transduction mechanisms as well as a variety of neurotrophic factors may be a common denominator to the detrimental consequences of chronic stress including induction and/or aggravation of neurodegenerative diseases as well as neuropsychiatric disorders including MDD.

## 6. Curcumin: Antidepressant

The above discussions have provided a framework for consideration of inflammation as an important player in the precipitation of depressive disorders. Hence, the well-documented anti-inflammatory effects of curcumin in *in vitro* and *in vivo* studies [107,108,109,110], including our own observation of its protective effects against LPS- and various cytokine-induced toxicity in SH-SY5Y cells [87] offer a mechanistic basis for observed antidepressant effects of curcumin. Indeed, numerous studies using various animal models of depression [14,15,16,111,112] including olfactory bulbectomized rat model of depression [113] as well as a recent report by us utilizing the WKY rats as a putative animal model of depression [19] provide solid evidence for an antidepressant effect of curcumin. Moreover, various clinical studies indicate therapeutic potential of curcumin in MDDs. These studies include randomized controlled trials of curcumin alone [114,115], or with piperine, an alkaloid found in black and long peppers, to boost the bioavailability of curcumin [116]. 

## 7. Curcumin: Neuroprotectant

Similarly, the role of neuro-inflammatory mediators as contributory factors to neurodegenerative diseases in general, and PD in particular, have been emphasized above. Numerous observations of the protective effects of curcumin against various inflammatory-mediated axonal or neuronal damage are all in line with its potential beneficial effects in treating neurodegenerative diseases including PD. For example, research shows its protection against: chronic inflammation associated with Alzheimer’s disease [117]; axonal degeneration from local neuroinflammation [118]; β-amyloid induced degeneration [119]; peripheral neuropathy [120]; experimental models of Huntington’s disease [121]; okadaic acid induced memory impairment in mice [122]; N-methyl N-nitrosourea induced functional and structural alterations in mice brain [123], as well as our own observation of its protective effects against damage induced by a combination of salsolinol and rotenone in neuroblastoma cell lines [87]. A survey of clinical studies on potential usefulness of curcumin in Alzheimer’s disease [124,125]; dementia [126] and PD further support its potential therapeutic benefits [127,128]. 

## 8. Conclusions

Based on the above discussion, it is not unreasonable to suggest that the anti-inflammatory properties of curcumin are at least partially responsible for its antidepressant and its neuroprotectant effects. However, a few important points have to be considered in this regard. The first point concerns the bioavailability of curcumin as dietary curcumin exhibits poor bioavailability [129]. This might be due to several factors including its insolubility in water, poor absorption and rapid metabolism that need to be overcome for a more meaningful therapeutic intervention with curcumin. In this regard, it has been suggested that addition of piperine could enhance its absorption [116,129]. Moreover, the bioavailability of curcumin; may be increased by its dissolution in oil or cooking [130]. This is key in understanding the effects seen in the epidemiological studies [24,25,26], as curcumin, piperine, and oil are often used in combination in traditional Indian cooking. However, pharmacological structural modification and development of novel curcumin derivatives that may be administered orally or intra-nasally may offer a more realistic and potent therapeutic approach. 

The other important point concerns possible combination of curcumin or its more stable derivatives with current or other medications to treat depression and/or PD. This approach may be particularly beneficial since different drugs may act at different sites and offer a better control of inflammatory and other processes (e.g., oxidation) that may contribute to the pathology of these diseases. In this regard, it is noteworthy that a number of novel compounds including nicotine, resveratrol, ketamine and more recently, estrogen receptor agonists, have shown potential usefulness in major depression and PD [131,132,133].

**Figure 1:**
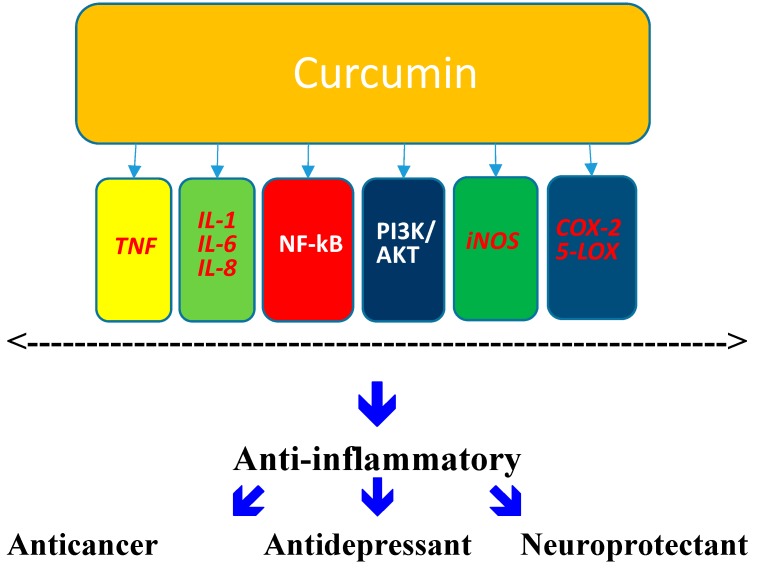
Potential Molecular Mechanisms Affected by Curcumin. The inhibition of cell signaling pathways such as Akt, NF-κB, or PI3K [139], has been suggested as the mechanism responsible for anticancer effects of curcumin. Curcumin may also inhibit the activity and synthesis of the enzymes implicated in inflammation such as cyclooxygenase-2 and 5-lipooxygenase. Moreover, its anti-inflammatory effect may be linked to inhibition of pro-inflammatory leukotrienes as well as to its neutrophil function [63,64]. Inhibition of TNF [59] may contribute to a physical interaction with other key signaling protein like BDNF that might enhance its antidepressant effect.

Finally, it is also of relevance to note that, in addition to the mechanisms mentioned above, curcumin might also affect a variety of other factors or mediators that have a direct role in neurodegenerative and/or mood regulating processes. To this end, it has been demonstrated that curcumin may affect: mitochondrial dysfunction [134]; oxidative stress [135]; molecular chaperones [136]; mTOR pathway [137] and MAPK expression [138]. Figure 1 provides some insight into few possible molecular mechanisms involved in neuroprotective and/or antidepressant effects of curcumin. These mechanisms warrant further research to understand their role in mediating the various actions that curcumin has in the brain.

In summary, novel compounds based on curcumin, alone or in combination with other known drugs that suppress inflammation and have been shown to exert antidepressant and/or neuroprotective effects may offer optimum therapy in major depression and PD.
